# Comparison of Traumatic Brain Injury in Adult Patients with and without Facial Fractures

**DOI:** 10.3390/tomography10100113

**Published:** 2024-09-24

**Authors:** Iulia Tatiana Lupascu, Sorin Hostiuc, Costin Aurelian Minoiu, Mihaela Hostiuc, Bogdan Valeriu Popa

**Affiliations:** 1 Clinical Emergency Hospital Bucharest, 014461 Bucharest, Romania; iulia_lupascu9e@yahoo.com (I.T.L.); costin.minoiu@umfcd.ro (C.A.M.); mihaela.hostiuc@umfcd.ro (M.H.); bogdanvaleriupopa@gmail.com (B.V.P.); 2 Department of Legal Medicine and Bioethics, Faculty of Dental Medicine, “Carol Davila” University of Medicine and Pharmacy, 050474 Bucharest, Romania

**Keywords:** traumatic brain injury, facial fracture, head trauma, facial injury, maxillofacial injury

## Abstract

Objectives: Facial fractures and associated traumatic brain injuries represent a worldwide public health concern. Therefore, we aimed to determine the pattern of brain injury accompanying facial fractures by comparing adult patients with and without facial fractures in terms of demographic, clinical, and imaging features. Methods: This single-center, retrospective study included 492 polytrauma patients presenting at our emergency department from January 2019 to July 2023, which were divided in two groups: with facial fractures (FF) and without facial fractures (non-FF). The following data were collected: age, sex, mechanism of trauma (road traffic accident, fall, and other causes), Glasgow Coma Scale (GCS), the evolution of the patient (admitted to a medical ward or intensive care unit, neurosurgery performed, death), and imaging features of the injury. Data were analyzed using descriptive tests, Chi-square tests, and regression analyses. A *p*-value less than 0.05 was considered statistically significant. Results: In the FF group, there were 79% (*n* = 102) men and 21% (*n* = 27) women, with a mean age of 45 ± 17 years, while in the non-FF group, there were 70% (*n* = 253) men and 30% (*n* = 110) women, with a mean age 46 ± 17 years. There was a significant association between brain injuries and facial fractures (*p* < 0.001, AOR 1.7). The most frequent facial fracture affected the zygoma bone in 28.1% (*n* = 67) cases. The most frequent brain injury associated with FF was subdural hematoma 23.4% (*n* = 44), and in the non-FF group, the most common head injury was intraparenchymal hematoma 29% (*n* = 73); Conclusions: Both groups shared similarities regarding gender, age, cause of traumatic event, and outcome but had significant differences in association with brain injuries, ICU admission, and clinical status.

## 1. Introduction

Traumatic brain injury (TBI), defined as an acquired form of brain damage due to external forces, represents a common cause of disability and death [1,2]. It was estimated that it affects over 10 million people every year, leading to either hospitalization or mortality [3].

The incidence and etiology of facial fractures vary significantly globally [4]. Some authors have claimed that approximately one in seven trauma patients presenting at the emergency department associate maxillofacial fractures [5], while others mention an incidence between 7.4% and 8.7% [6]. Older studies have suggested that facial fractures are associated with a lower risk of traumatic brain injury due to facial bones working as cushions by absorbing the energy of the impact [7]. Other studies, however, concluded that facial bone fractures do not help prevent traumatic brain injury and are, in fact, a marker for increased risk of head injury [8,9].

The most common causes of maxillofacial fractures reported are road traffic accidents (RTA), followed by falls from height [10,11,12], with a predominance of the male gender [13,14,15]. High-energy trauma is likely to cause concurrent facial and head injury in comparison with trauma with low energy. This variation in trauma mechanisms can influence the types and frequencies of the observed injuries [16].

Routine clinical imaging for the evaluation of TBI consists of non-contrast multi-detector CT (MDCT) and magnetic resonance imaging (MRI) for selected cases. MDCT has become the primary choice since it can detect fractures, foreign bodies, hemorrhage, intra- and extra-axial lesions. However, MRI has shown superior sensitivity for detecting diffuse axonal injuries and minor contusions [17,18]. Noninvasive angiography (CT angiography or MR angiography) may be required to describe vascular axes in cases of vascular abnormality [18,19].

The incidence of brain injury accompanying facial fractures is highly variable, ranging from 5.4% to 87% [10]. Pappachan et al. reported that mid-facial fractures are highly associated with traumatic brain injury [20]. Plaisier et al. stated that patients with any facial fracture involving the midface or upper face had a 13.5-fold increase in death compared with isolated lower facial fractures [21]. Tung et al. noticed that intracranial hematoma was the most common life-threatening injury in facial fracture patients [22]. Even though it has been noticed that facial fractures lead to a 1.5–2.4 times greater risk of brain injury compared with the non-facial fracture group, there is a lack of data on the comparison of brain injury patterns among the two groups [23,24]. In addition, there is also scarce and conflicting information in the literature about the precise relationship between different types of facial fractures and head injury patterns [25].

The fact that the brain is encased in a skeletal framework and patients can present initially with mild symptoms makes detection of brain injuries difficult. The heterogeneity of brain lesions contributes to the difficulty of their management. Therefore, identifying etiology and determining severity and distribution of TBI associated with facial fractures are essential for neurosurgeons and maxillofacial surgeons to develop optimal treatment strategies [26].

Our study aimed to determine the pattern of brain injury accompanying facial fractures by comparing adult patients with and without facial fractures in terms of demographic, clinical, and imaging features.

## 2. Materials and Methods

This study was performed with the approval of the Ethics Institutional Review Board, under the no. 9525/17 October 2023.

We performed a retrospective study on polytrauma patients at the emergency department of Clinical Emergency Hospital Bucharest from January 2019 to July 2023. We used the General Electric CT Optima 660 (GE Healthcare, Milwaukee, WI, USA) with 128 slices. The Cerebral CT scan included bone and parenchymal windows, with a slice thickness of 1.25 mm or 2.5 mm. Reformatted images in the sagittal and coronal planes were obtained for most patients. Images were analyzed independently by two physicians.

Among the inclusion criteria were age over 17 and polytrauma patients, defined as patients with at least two injuries who had at least a non-contrast Cerebral CT performed. The exclusion criteria were patients without a Cerebral CT performed and patients with CT performed in another medical unit.

The complete medical record of each patient fulfilling the inclusion criteria was reviewed. The following data were collected using Microsoft Excel software (last version no. 2408): age, sex, mechanism of trauma (road traffic accident, fall, and others (in which we included less frequent causes such as occupational accident, assault, tractor accident, or “not informed”), GCS, evolution of the patient (admitted to medical ward or intensive care unit, craniotomy performed, death), and imaging features of the injury on CT. Patients were separated into two groups: with facial fractures (FF) and without facial fractures (non-FF).

The maxillofacial fractures were divided based on location: frontal sinus fracture, nasal fracture, maxillary fracture, zygomatic fracture, and mandibular fracture. Calvaria and skull base fractures were considered as one entity.

Brain injuries evaluated were subdural hematoma, epidural hematoma, subarachnoid hemorrhage, intraventricular hemorrhage, intraparenchymal hemorrhage, pneumocephalus, diffuse brain swelling, and brain herniation.

Brain herniation was considered subfalcine if the middle line shifts more than 5 mm. Intracranial hemorrhagic lesions were defined as both intra-axial and extra-axial lesions, consisting of intraparenchymal hematoma, subdural, epidural hematoma, subarachnoid hemorrhage, and intraventricular hemorrhage.

Both facial fractures and brain injuries were evaluated qualitatively as a dichotomous variable yes/no. No radiomics features were extracted from CT images.

Based on GCS, patients were divided into three groups: group I: 3–8 score, accounting for severe head injury; group II: 9–12 score, representing moderate head injury; and group III: 13–15 score, mild head injury.

The SPSS version 26 (Statistical Package for Social Sciences) software program was used for statistical analyses. According to the type of data, the following tests were used: descriptive tests, Chi-square tests, and regression analyses. Missing data were managed through case deletion. A *p*-value of less than 0.05 was considered statistically significant.

## 3. Results

**Figure 1:**
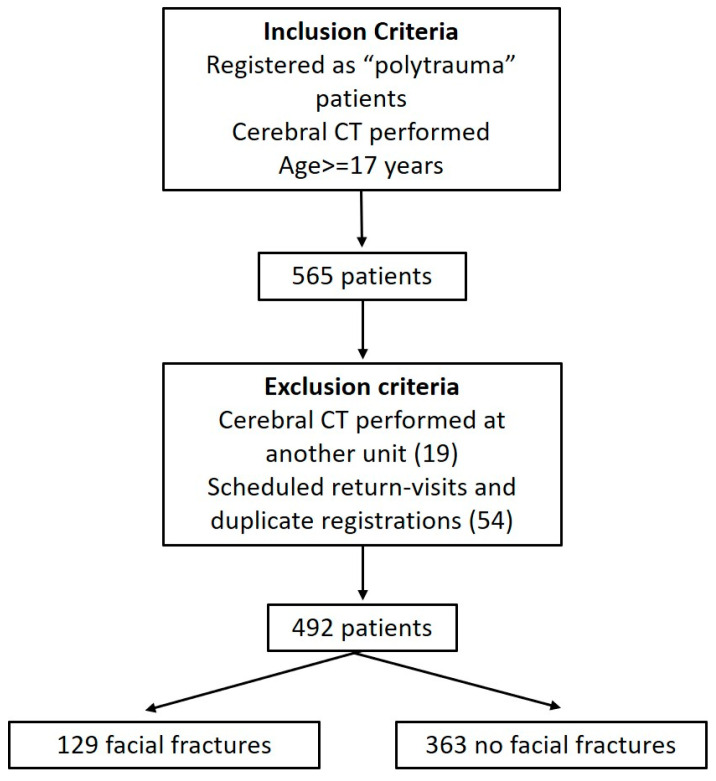
Flowchart of the inclusion process. This shows the inclusion and exclusion criteria, the number of patients that were excluded in each step, and the final number of patients.

We included 492 subjects, of which 26.2% (*n* = 129) subjects had FF and 73.8 (*n* = 363) were non-FF (Figure 1). The patient demographic profile revealed that in the FF group, there were 79% men (*n* = 102) and 21% women (*n* = 27), with ages ranging from 17 to 89, and a mean age 45 ± 17 years, while in the non-FF group, there were 70% (*n* = 253) men and 30% (*n* = 110) women, with ages ranging from 17 to 92, and a mean age 46 ± 17 years (Table 1). No significant association has been found between facial fractures and the gender or age of subjects in both groups.

Road traffic accidents were the leading cause of trauma in both groups, with 78% (*n* = 86) cases in the FF group, followed by falls 16% (*n* = 18) and other causes 6% (*n* = 6), while in the non-FF group, road traffic accidents were the cause of trauma in 75% (*n* = 262) cases, followed by falls 16% (*n* = 56) and other causes 8% (*n* = 29).

Overall, 81% of the subjects with FF (*n* = 101) and 70.7% (*n* = 253) without FF needed ICU admission. FF has been significantly correlated with ICU admission (*p* < 0.05)

Brain injuries were identified in 57% (*n* = 71) of subjects with FF and 32% (*n* = 115) subjects without facial fractures, among which intracranial hemorrhagic lesions were found in 54% (*n* = 68) subjects from the FF group and in 31% (*n* = 111) subjects from the non-FF group.

Forty-two percent (*n* = 51) of subjects had only one facial bone fractured; the rest presented with at least two facial bones fractured.

**Figure 2:**
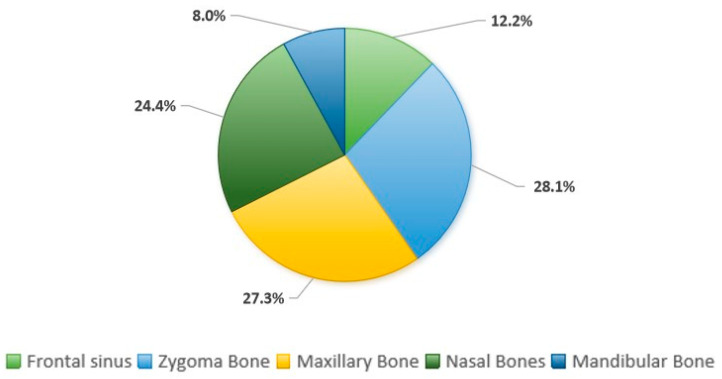
Prevalence of facial fractures.

The most frequent facial fractures affected the zygoma bone in 28.1% (*n* = 67) cases, followed by the maxillary 27.3% (*n* = 65), nasal bones 24.4% (*n* = 58), frontal sinus 12.2% (*n* = 29), and the mandible in 8% (*n* = 19). For details, see Figure 2. 

Overall, 19.8% (*n* = 96) of all subjects had calvaria and skull base fractures. From the FF group, 40% (*n* = 50) had calvaria and skull base fractures, and from the non-FF group, 13% (*n* = 46).

**Figure 3:**
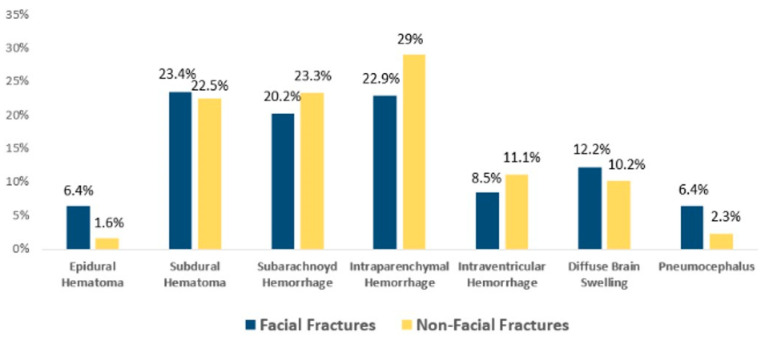
Types of brain injury in both FF and non-FF group and their prevalence.

The most frequent brain injury associated with FF was subdural hematoma accounting for 23.4% (*n* = 44); followed by intraparenchymal hematoma, 22.9% (*n* = 43); subarachnoid hemorrhage, 20.2% (*n* = 38); diffuse brain swelling, 12.2%; intraventricular hemorrhage, 8.5% (*n* = 16); epidural hematoma, 6.4% (*n* = 12); and pneumocephalus, 6.4% (*n* = 12). In the non-FF group, the most common brain injury was intraparenchymal hematoma 29% (*n* = 73); followed by subarachnoid hemorrhage, 23.3% (*n* = 59); subdural hematoma, 22.5% (*n* = 57); intraventricular hemorrhage, 11.1% (*n* = 28); diffuse brain swelling, 10.5% (*n* = 27); pneumocephalus, 2.3% (*n* = 6); and epidural hematoma, 1.6% (*n* = 4). (Figure 3)

We identified significant correlations between frontal sinus fracture and epidural hematoma (*p* < 0.01, OR: 6.1), between zygoma fracture and subdural hematoma (*p* < 0.05, OR: 2.7), between zygoma fracture and diffuse brain swelling (*p* < 0.05, OR: 3.1), between zygoma fracture and pneumocephalus (*p* < 0.05, OR: 5.1), and between maxillary bone fracture and brain herniation (*p* < 0.05).

The location of the frontal bone fracture was significantly associated with the location of epidural hematoma (*p* < 0.01) and intraparenchymal hematoma (*p* < 0.01); the zygoma fracture location was related to the location of subdural hematoma (*p* < 0.01) and subarachnoid hemorrhage (*p* < 0.01); and maxillary bone fracture location with location of epidural hematoma (*p* < 0.01), subdural hematoma (*p* < 0.01), intraparenchymal hematoma (*p* < 0.05), and subarachnoid hemorrhage (*p* < 0,01).

Zygoma fracture was the most correlated with brain injuries in 32% (*n* = 44) cases; followed by maxillary bone, 29% (*n* = 40); nasal bones, 18% (*n* = 25); frontal bone, 14% (*n* = 20); and mandibular bone, 7% (*n* = 10). 

We identified severe head injury in 50% (*n* = 41) of the subjects with FF, moderate head injury in 9% (*n* = 7), and mild head injury in 41% (*n* = 33).

In the non-FF group, severe head injury was found in 44% (*n* = 102) cases, moderate head injury in 5% (*n* = 12), and mild head injury in 51% (*n* = 120). In both groups, the risk of head injury increased as the GCS score decreased.

Overall, 51% (*n* = 41) of the subjects with a severe head injury in the FF group and 43% (*n* = 100) with a severe head injury from the non-FF group required an ICU stay. Admission to ICU was associated with GCS (*p* < 0.01) in both groups.

Most subjects with a severe GCS score were associated with maxillary bone fracture, 32% (*n* = 23); followed by zygoma, 28% (*n* = 23); frontal, 16% (*n* = 11); nasal, 14% (*n* = 10); and mandibular bone, 10% (*n* = 7). 

Subjects with a severe score in FF group had subdural hematoma as the most frequent head lesion, 22.7% (*n* = 25); followed by subarachnoid hemorrhage, 21.8% (*n* = 22); intraparenchymal hemorrhage, 18% (*n* = 20); diffuse brain swelling, 15.5% (*n* = 14); pneumocephalus, 9.1% (*n* = 10); intraventricular hemorrhage, 9.1% (*n* = 10); and epidural hematoma, 4.5% (*n* = 5). 

In the non-FF group, GCS severe score subjects had intraparenchymal hemorrhage as the most frequent head injury in 25.5%(*n* = 40) subjects, followed by subarachnoid hemorrhage in 24% (*n* = 38), subdural hematoma in 22% (*n* = 35), diffuse brain swelling in 14% (*n* = 21), intraventricular hemorrhage in 11.5% (*n* = 18), pneumocephalus in 2.5% (*n* = 4), and epidural hematoma in 0.5% (*n* = 1).

Neurosurgical decompression was performed in 8.8% (*n* = 11) cases with FF and 2% (*n* = 7) without FF, and it was significantly correlated with the presence of brain injuries (*p* < 0.001). Middle line shift was identified in 6% (*n* = 8) cases from the FF group and 2% (*n* = 6) cases from the non-FF group.

**Figure 4:**
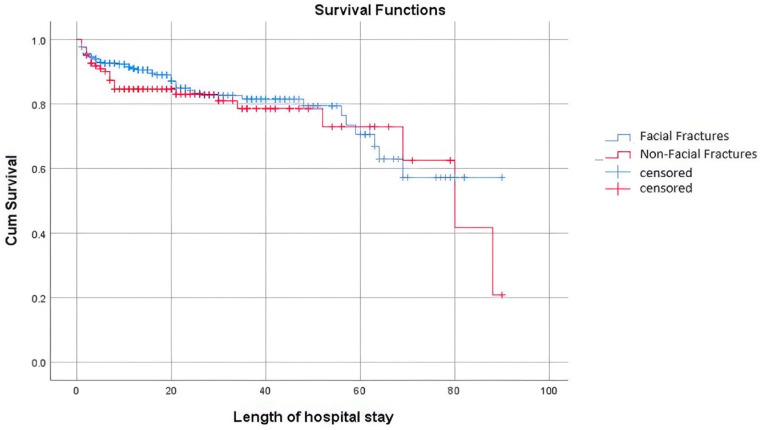
Kaplan–Meier survival curve for length of stay in the hospital.

The mean length of stay in the hospital for the FF and non-FF group was 23 ± 21 days and 22 ± 19 days, respectively, and there was no significant difference between the groups. Kaplan–Meier survival curves for length of stay in the hospital are shown in Figure 4.

Overall, 20% percent (*n* = 25) of subjects with FF and 14.5% (*n* = 52) without FF died. Death was correlated with GCS and the presence of brain injuries among both groups (*p* < 0.01). The association between death and brain injuries was stronger for the FF group (*p* < 0.001; OR: 11.4). 

In the FF group, 17% (*n* = 22) of patients with brain injury died, while in the non-FF group, 8% (*n* = 30) of patients with brain injury died. In both groups, the presence of brain injury was significantly correlated to death (*p* < 0.001; OR:11.4 for FF and *p* < 0.001; OR:3.6 for non-FF). The non-FF group had a significantly decreased mortality compared associated with the presence of brain injuries (OR = 0.348, 95%CI: 0.14–0.41). In the FF group, we found significant associations between death and subdural hematoma (*p* < 0.001; OR: 5.8), subarachnoid hemorrhage (*p* < 0.001; OR: 8.7), intraparenchymal hemorrhage (*p* < 0.001; OR:5.8), intraventricular hemorrhage (*p* < 0.001; OR: 4.8), diffuse brain swelling (*p* < 0.001; OR:8.7), brain herniation (*p* < 0.05; OR: 7.5), and pneumocephalus (*p* < 0.05; OR: 6.9). In the non-FF group, we found a significant association between death and subdural hematoma (*p* < 0.001; OR: 3.7), subarachnoid hemorrhage (*p* < 0.001; OR: 4.7), intraparenchymal hemorrhage (*p* < 0.001; OR: 2.4), intraventricular hemorrhage (*p* < 0.001; OR: 6.4), diffuse brain swelling (*p* < 0.001; OR: 11.9), brain herniation (*p* < 0.001; OR: 13.2), and pneumocephalus (*p* < 0.05; OR: 6.2). 

In the FF group, there were no deaths in mild GCS scores, 1.2% (*n* = 1) deaths in moderate scores, and 22%(*n* = 18) deaths in severe scores, while in non-FF group, there were 0.5% (*n* = 1) deaths in mild GCS scores, 0.8% (*n* = 2) in moderate, and 17% (*n* = 39) in severe scores.

In this study, we found a significant association between brain injuries and facial fractures (*p* < 0.05; AOR: 1.7).

## 4. Discussion

In this study, we aimed to evaluate the relationship between facial fractures and TBI while comparing patients with and without facial fractures in terms of demographic, clinical, and imaging features based on CT evaluation in acute settings. We showed that both groups share similarities regarding gender, age, cause of traumatic event, and outcome but significant differences in association with brain injuries, ICU admission, neurosurgery, and clinical status. 

The need to conduct this study was based on the fact that facial fractures and concurrent TBI represent a worldwide public health concern. Even though many studies have investigated their relationship in the past decades, the results are very heterogenous, and this leads to difficulties in implementing them in guidelines/recommendations [25]. Moreover, there is a low number of studies comparing patients with and without facial fractures [27,28], which was the aim of this study. 

The clinical relevance of the study stems from how important it is for radiologists, craniofacial surgeons, and trauma specialists to know what to expect in an emergency setting when assessing patients who have sustained facial fractures. Being aware of the association between facial fractures and brain injury can lead to paying more attention to small lesions that otherwise might be overlooked during a night shift.

Currently, MDCT is the primary choice of initial evaluation of TBI, as it is readily available for the majority of trauma centers and emergency rooms. It has replaced single-detection systems since it allows the acquisition of multiple thin sections in a shorter time. Another advantage is the reconstruction of image data in every plane or 3D view. Multiplanar reconstructions (MPR) have been shown to improve the diagnosis of traumatic injury and increase CT accuracy. CT angiography can be performed when there is a suspicion of vascular injury and in patients with high-risk trauma mechanisms such as high-energy crashes, fractures of the midface and skull base, and near-hanging or intra-oral trauma [17]. Post-mortem CT also plays an important role, highlighting the presence of fractures, foreign bodies, or vascular lesions, such as posttraumatic aneurysms, an aspect which is useful in traffic accidents [29,30]. However, CT offers only a macroscopic view of the brain, without capturing changes such as diffuse axonal injury. Furthermore, an initial CT with no lesions does not exclude a clinical injury, such as delayed hemorrhage, which can show up even several weeks after the initial traumatic moment [31].

On the other hand, MRI is more sensitive for the detection of both diffuse axonal injury and small non-hemorrhagic contusions. Still, it is not typically used for the initial evaluation of TBI since the acquisition takes a longer time, is less available in hospitals, and requires safety screening for metallic foreign bodies or incompatible medical devices [18]. It can also be indicated when there is a discrepancy between CT findings and the patient’s neurological status [17]. Our study included patients evaluated only using CT since MRI was not available in the acute setting, an aspect which represents a limitation.

In this study, most subjects were male, with 79% in the FF group and 70% in the non-FF group. The male-to-female ratio in the FF group was approximately 4:1, comparable with most other studies in the area, which showed a range varying from 2.6:1 to 11.8:1 [27]. A possible explanation for this is that men tend to engage more often in high-risk activities and dangerous behaviors, being therefore more vulnerable to accidents [32,33].

In our study, most facial fractures were caused by road traffic accidents (both motor vehicles and motorcycles) and falls. This etiology is consistent with most studies in this area. You et al. reported motor vehicle crashes to be the most prevalent mechanism of injury (40.3%), followed by falls (26%); Nawi et al. mentioned motorcycle accidents to be the most common (50.7%); Elbaih et al. reported 53.3% of polytrauma patients with facial fractures had motor car accidents [25,32,34]. These findings should raise concern regarding the enforcement of road traffic safety regulations that include wearing seatbelts and helmets, speed limitations, alcohol consumption, and educating citizens, who can face lifelong permanent neurological disabilities [10,23]. However, it is worth mentioning that other studies have reported assault as the main etiology of injury (41%) [35]. Gomez Rosello et al. also mentioned assault to be the most prevalent mechanism of injury (44–61%) [36]. It is thought that road traffic accidents represent the main cause of traumatic injury in most developing countries, while violence occurs mainly in developed regions such as North America and Europe [26].

Identifying the etiology is essential since variations in trauma mechanisms can influence the types and frequencies of the injuries. In general, high-velocity and high-energy impacts to the upper third of the face such as motor vehicle accidents are the leading cause of frontal sinus fracture, an incidence that might have reduced with the use of airbags. Other causes remain impact sports such as boxing, extreme sports, but also falls from heights or industrial accidents [37].

Nawi et al. mentioned the category of 11–20 years old as the most prevalent one for patients with facial fractures, while Elbaih et al. and Joshi et al. noticed most patients with facial fractures and concomitant TBI belonged to the 10–30-years-old group, followed by the 30–50-years-old group [10,25,32]. Hwang and Kim reported the highest frequency of facial bone fractures being in the 21–30 years age group [38]. In our study, most subjects belonged to the 30–45-year-old age category in both groups. A possible explanation for these different results is that our hospital provides treatment and services for adults only; therefore, our study did not have patients below the age of 17. Another explanation is related to differences in countries’ demographic data, since in Romania, the median age is 41.4, according to DataReportal 2023 [39].

We found no significant association between brain injury and the age of the patients. This aspect is not following Joshi et al. or Lee et al. [7,10]. However, other studies support it, where no significant association between head injury and age was found [40]. We also found no significant correlation between brain injury and the gender of patients in both groups. 

Fifty-seven percent of patients with facial fractures in our study also showed brain injury. In other studies, the rate of brain injury concurrent with facial trauma showed a high variation from 5.4% to 86% [10,24,41,42]. This variation might be due to methodological differences among studies, different selection criteria, and a lack of a proper definition for “brain injury” [10,41].

In a study by Lee et al., it was found that facial fractures presented a lower risk of traumatic brain injury, as facial architecture offered cushion-like protection for the brain [7]. Other studies, however, did not support this theory, mentioning that facial injuries increased the risk of intracranial injury almost 10-fold [8]. Our results agree with the latter studies, as we found that facial fractures had a 1.7 times higher significant risk of being associated with TBI and a 4.8 times greater need of neurosurgical decompression. Isik et al. observed that the risk of brain injury increases in patients with multiple facial bone fractures compared with single facial bone fractures [43]. These results have clinical relevance, suggesting the need for emergency computed tomography to prevent morbidity and mortality associated with TBI [19].

Our study showed that most patients with FF (81%) needed ICU admission, which is similar with the results presented by Zandi and Seyed Hosini, where 74% of FF patients were admitted to ICU for varying duration [24]. We found that the presence of FF was significantly associated with ICU admission.

High-energy trauma, which causes facial fractures, can lead to rupture of intracranial vessels and, therefore, can lead to intracranial hemorrhage in various compartments. Therefore, the injuries accompanying facial trauma represent a more significant threat than the fracture itself [44]. In our study, 54% of patients with FF showed intracranial hemorrhage, which represents a higher percentage compared to Kanno et al., who reported an incidence of only 9% [44]. The different methods of patient selection can explain such a discrepancy; our study included polytrauma patients, possibly engaged in higher-energy trauma.

Regarding the type of traumatic brain injury, this study revealed that the majority of FF patients presented with subdural hematoma, followed by intraparenchymal hemorrhage, while a minority of patients presented with pneumocephalus and epidural hematoma. In the non-FF group, the most prevalent head injury was intraparenchymal hemorrhage, followed by subarachnoid hemorrhage, and the least encountered injuries were also pneumocephalus and epidural hematoma. Alvi et al. also mentioned subdural hematoma to be the most common intracranial bleeding in patients with facial fractures, representing 41.3% of all cerebral bleeds [35].

Regarding the most common site of facial fracture, the zygoma bone was the most prevalent one, followed by the maxillary bone, which was reported by Mao et al. [26]. Rajendra et al. reported that the most commonly fractured bone was the zygoma followed by the mandible and maxillary [45]. Hwang and Kim mentioned nasal bones fractures to be the most common, followed by mandible bone fractures; Carvalho et al. mentioned mandible fractures as most frequent, followed by nasal bones fractures; while Alvi et al. reported orbital fractures to be the most encountered ones [6,35,38]. These conflicting results can result from variations in the methodology, classification, and nomenclature of previous studies [24]. 

As far as the connection between facial fractures and concurrent brain injury is concerned, in our study, zygoma fractures were most commonly associated with brain injuries, having a statistically significant relationship with subdural hematoma, diffuse brain swelling, and pneumocephalus. Following zygoma fracture, the most likely to be associated with TBI were maxillary and nasal bones fractures.

The facial bone least associated with TBI in our study was the mandibular bone. A possible reason for this is that the mandible is anatomically distant from the cranium and suspended below the maxilla through muscles and ligaments, without osseous connection, which might act as a cushion against force transmission towards the cranium, therefore being less likely to accompany brain injuries [26]. This result is similar to other studies, such as McCarty et al., who reported that brain injury in patients with isolated facial fractures was lowest in isolated mandibular fractures and increased going up the craniofacial skeleton, and Kloss et al., who stated that zygoma and orbit were the most common fractured bones associated with intracranial hemorrhage [40,46]. This is also supported by our findings, where most patients with a severe score of GCS were associated with maxillary bone fracture followed by zygoma fracture, while the mandibular bone fracture was the least associated with a severe GCS. 

Regarding correlations between fracture sites and TBI, we identified significant associations between frontal sinus fracture and epidural hematoma, between zygoma fracture and subdural hematoma, and between pneumocephalus and diffuse brain swelling, maxillary bone fracture, and brain herniation. Regarding the location of the fracture, frontal sinus fracture was significantly associated with the location of epidural hematoma and intraparenchymal hematoma; the zygoma fracture location was associated with the location of subdural hematoma, subarachnoid hemorrhage, and maxillary bone fracture location with the location of epidural, subdural, intraparenchymal hematoma, and subarachnoid hemorrhage. 

In our study, 8.8% of patients with FF had neurosurgery, a slightly higher percentage than in other studies. Kanno et al. reported that 2.1% of patients with maxillofacial fractures had neurosurgery performed [44]. A possible explanation for this difference is the method of patient selection since our study included polytrauma patients. 

Regarding the severity of the head injury, most subjects in the FF group (50%) had low GCS scores (<8), while in the non-FF group, mild scores (>13) were the most common (51%). Some studies, however, have stated that mild head injury is the most prevalent among patients with facial fractures, followed by severe head injury [25,32]. This distinction might result from different patient selection criteria, as our study was focused only on polytrauma patients. Nordin et al. reported 76.9% of their TBI were mild, and Abdul Razak et al. reported 41.4% of patients with facial injuries had mild TBI [47,48]. Even so, CT abnormalities can be found in mild TBI, proving that a high GCS score does not necessarily mean a lack of brain injury. Borczuk noticed abnormal computed tomography findings in 119 out of 1448 patients with GCS scores of 13–15 [49]. Another study, including patients with mild TBI who had a CT scan performed in the emergency department, found 11.6–16.1% patients with mild TBI had associated an intracranial lesion [50].

In our study, in both groups, there was a significant association between GCS and brain injury, with the risk of head injury increasing as GCS decreased, an aspect that correlates to other studies in the literature [10]. Most of our patients from the FF group who had a severe head injury were admitted to the ICU. We found a significant association between the ICU and GCS. However, even though the GCS is sensitive to neurologic injury, it is not specific. Early sedation and intubation are viewed as factors that impact GCS assessment and represent a problem in obtaining a neurological evaluation in the first 24 h after trauma [51].

The mean length of stay in the hospital for patients with FF was 23 ± 21 days, with no significant difference from the non- FF group. Other studies have reported an average hospital stay of 9.6 days, varying from 1 to 39 days [35]. Hwang and Kim reported an average hospital stay of 8.4 days, while Holmgren et al. mentioned an average of 7.8 days [28,38]. This can be influenced by the selection criteria, type of hospital, and associated injuries, which can act as confounders. 

Twenty percent of our patients with FF died in the hospital. We did not notice any significant association between death and a particular type of facial fracture. However, some studies have mentioned a strong correlation between patterns of upper face or midface fractures and death of neurologic injury, with a 13.5-fold increase in neurologic death in the “assume the worst” scenario in these patients compared to patients with an isolated lower facial fracture [21].

Some studies have mentioned a hospital mortality of 8.6% of patients with FF, while others have reported a rate of 2.2% [35,52]. The main reasons reported for death in patients with facial fractures are asphyxia because the upper airways have been compromised or massive bleeding from the major vessels of the head and neck; these factors account for 24% of the deaths of patients involved in motor vehicle accidents [52].

In our study, patients with facial fractures were associated with a higher ICU admission rate, brain injury rate, higher need for neurosurgical decompression, and mortality in comparison with patients without facial fractures. Our results agree with a study comparable to ours conducted by Holmgren et al. [28]. However, we did not notice differences between the groups in regard to gender or length of hospital stay, which results in contradiction with the study above. A possible explanation for these differences is related to the fact that our study included polytrauma patients in both groups, with potential thoracic-abdominal-pelvic injuries, which also influence the length of hospital stay for both facial fractures and non-facial fractures groups.

## 5. Limitations

Due to the retrospective nature of the study, we are missing information regarding the patients’ follow-up, so it was not possible to determine long-term consequences or the actual mortality rate. We also have incomplete data regarding the use of seatbelts or helmets, alcohol consumption, or substance use. The lack of MRI use in the study represents a limitation, as we did not evaluate patients for diffuse axonal injuries. We also did not evaluate in this study associated lesions, such as thoraco-abdomino-pelvic injuries, that can act as confounders. These variables, alongside biomarkers and preinjury pathologies, should be considered in future research for a better understanding. Since our study involved only one trauma center, the generalization of the results is reduced. However, our results agree with other studies and are statistically significant.

## 6. Conclusions

Our study showed that facial fractures were significantly associated with higher rates of brain injury, ICU admission, need for neurosurgical decompression, and death in the hospital compared to patients without facial fractures. Most patients with concurrent facial fractures and TBI showed a low GCS score (<8) at the time of admission compared to patients without facial fractures, among which higher scores (13–15) were the most common. These results indicate the importance of quick diagnosis, early intervention, and the need for the routine use of head CT to lower the risk of morbidity and mortality associated with brain injury. Our study contributes to the patterns of traumatic brain injuries related to facial fractures for radiologists, craniofacial surgeons, and trauma specialists to know what to expect in an emergency setting.

## Figures and Tables

**Table 1:** Main data of patients.

	Facial Fractures	Non-Facial Fractures
Patients	129 (26.2%)	363 (73.8%)
Gender		
Male	102 (79%)	253 (70%)
Female	27 (21%)	110 (30%)
Age		
<30	27 (21%)	85 (24%)
30–45	43 (33%)	98 (27%)
46–60	33 (26%)	92 (25%)
61–75	21 (16%)	69 (19%)
>75	5 (4%)	19 (5%)
Mean	45 ± 16	46 ± 16
Range	17–89	17–92
Main cause	RTA	RTA
RTA	86 (78%)	262 (75%)
Fall from heights	18 (16%)	56 (16%)
Other causes	6 (6%)	29 (8%)
ICU admission	101 (81%)	253 (70,7%)
GCS mild	33 (41%)	120 (51%)
GCS moderate	7 (9%)	12 (5%)
GCS severe	41 (50%)	102 (44%)
Brain injury	71 (57%)	115 (32%)

RTA: road traffic accidents; GCS: Glasgow Coma Scale.

## Data Availability

The raw data supporting the conclusions of this article will be made available by the authors on request.
